# Supplement Treatment with NAC and Omega-3 Polyunsaturated Fatty Acids during Pregnancy Partially Prevents Schizophrenia-Related Outcomes in the Poly I:C Rat Model

**DOI:** 10.3390/antiox12051068

**Published:** 2023-05-09

**Authors:** Henriette Edemann-Callesen, Nadine Bernhardt, Elizabeth Barroeta Hlusicka, Franziska Hintz, Bettina Habelt, Rebecca Winter, Isabell Neubert, Meike Pelz, Alexandra Filla, Maria Luisa Soto-Montenegro, Christine Winter, Ravit Hadar

**Affiliations:** 1 Department of Psychiatry and Neuroscience, Campus Mitte, Charité University Medicine Berlin, Charitéplatz 1, 10117 Berlin, Germany; au274414@uni.au.dk (H.E.-C.);; 2 Department of Psychiatry and Psychotherapy, Medical Faculty Carl Gustav Carus, Technische Universität, 01307 Dresden, Germany; 3 Leibniz Institute of Polymer Research Dresden, 01069 Dresden, Germany; 4 Instituto de Investigación Sanitaria Gregorio Marañón, 28007 Madrid, Spain; 5 CIBER de Salud Mental, Instituto de Salud Carlos III, 28029 Madrid, Spain; 6 Grupo de Investigación de Alto Rendimiento en Fisiopatología y Farmacología del Sistema Digestivo (NeuGut-URJC), Universidad Rey Juan Carlos, 28922 Alcorcón, Spain

**Keywords:** N-acetyl cysteine, schizophrenia, omega-3 polyunsaturated fatty acids, preventive treatment, supplements

## Abstract

Background: Heightened levels of inflammation and oxidative stress are thought to be involved in the pathophysiology of schizophrenia. We aimed to assess whether intake of anti-inflammatory and anti-oxidant drugs during pregnancy prevents later schizophrenia-related outcomes in a neurodevelopmental rat model of this disorder. Methods: Pregnant Wistar rats were injected with polyriboinosinic–polyribocytidilic acid (Poly I:C) or saline and subsequently treated with either N-acetyl cysteine (NAC) or omega-3 polyunsaturated fatty acids (PUFAs) until delivery. Controls rats received no treatment. In the offspring, neuroinflammation and anti-oxidant enzyme activity were assessed on postnatal day (PND) 21, 33, 48, and 90. Behavioral testing was performed at PND 90, followed by post-mortem neurochemical assessment and ex vivo MRI. Results: The supplement treatment led to a quicker restoration of the wellbeing of dams. In the adolescent Poly I:C offspring, the supplement treatment prevented an increase in microglial activity and partially prevented a deregulation in the anti-oxidant defense system. In the adult Poly I:C offspring, supplement treatment partially prevented dopamine deficits, which was paralleled by some changes in behavior. Exposure to omega-3 PUFAs prevented the enlargement of lateral ventricles. Conclusion: Intake of over-the-counter supplements may assist in especially targeting the inflammatory response related to schizophrenia pathophysiology, aiding in diminishing later disease severity in the offspring.

## 1. Introduction

Schizophrenia is a neurodevelopmental disorder in which a progressive accumulation of underlying neuropathological processes eventually results in the manifestation of symptoms later in life [1,2]. The initiation of such processes is likely a result of interacting genetic and environmental risk factors, of which some of the latter may involve ongoing heightened levels of inflammation and oxidative stress, presumably set in motion by early immune dysregulation [2,3,4,5,6]. Accumulating evidence indicates that early systemic inflammation and oxidative stress play a central role the pathophysiology of schizophrenia, as the disruptive nature of such processes has been linked to cardinal features of the disorder, such as alterations in both the myelination of neuronal circuits and expression of parvalbumin GABAergic interneurons [7,8]. In line with this, anti-inflammatory and anti-oxidant drugs are suggested as potential treatment strategies [9,10,11]. Different drugs have been investigated including vitamin C, vitamin E, Ginkgo biloba, omega-3 polyunsaturated fatty acids (PUFAs), and N-acetyl cysteine (NAC). Of these, especially Gingko biloba, NAC, and omega-3 PUFAs have shown promising results, yet further studies are needed [12,13,14,15]. For omega-3 PUFAs in particular, low levels have been found in the orbitofrontal cortex as well as in the erythrocyte membranes in patients with schizophrenia [16,17]. Treatment with omega-3 PUFA supplements have furthermore shown positive effects in the early stages of schizophrenia, whereas a smaller effect was seen in chronic patients, indicating that timing of the treatment is important [12,18]. 

From a developmental point of view, it is of interest whether intake of drugs with anti-oxidative and anti-inflammatory properties may counteract the later development of schizophrenia-related outcomes if they are applied early in development before the immune dysregulation is set in motion. As prenatal inflammation is considered a risk factor of schizophrenia, preventive measures may already be needed at the fetal stage for high-risk individuals [19,20]. Both NAC and omega-3 PUFA supplements are considered safe during pregnancy as well as beneficial for both mother and child. Omega-3 PUFAs are generally recommended in pregnancy if they are not sufficiently provided through the diet, due to its benefits on neurodevelopmental outcomes in the offspring [21]. NAC is suggested as a supplement for high-risk pregnancies, as it may decrease the risk of preterm delivery and improve neonatal outcomes [22,23]. Gingko biloba, however, should be used with caution during pregnancy [24].

The Poly I:C rat model is a neurodevelopmental model of schizophrenia based on prenatal exposure to a virus-mimicking compound, which leads to a protracted development of schizophrenia-like behaviors in the offspring similar to those described in patients [25,26]. It has previously been shown that behavioral and associated neurobiological alterations in the Poly I:C rat model may be prevented if interventions are given prior to symptom manifestation [27,28,29,30]. Different preventive avenues that have been applied to adolescent Poly I:C animals have proven beneficial, including the use of antipsychotics, hormonal agents, different brain stimulation techniques, as well as NAC and omega-3 PUFAs [29,31,32,33,34,35,36]. 

As the neurobiological trajectories in the Poly I:C rat model are already observed prenatally, our aim was to investigate whether prenatal supplement treatment with omega-3 PUFAs or NAC may prevent later schizophrenia-related outcomes in the offspring of the Poly I:C rat model. 

## 2. Materials and Methods

### 2.1. Animals

All procedures were performed in accordance with the European Communities Council Directive of 22 September 2010 (2010/63/EU) and after approval by the local ethics committee (Landesdirektion Sachsen, TVV 15/2016). Female pregnant Wistar rats (*n* = 48) were obtained from Charles River Laboratories, Europe. The dams were housed individually until delivery. On postnatal day (PND) 21, the offspring were weaned and housed in groups of 2–4 animals in a temperature- and humidity-controlled vivarium under a 12 h light standard day cycle (lights on at 6 a.m.) with food and water provided ad libitum (unless otherwise stated). All efforts were made to reduce and avoid the animals’ suffering and minimize the number of animals used.

### 2.2. Experimental Design

The project followed a 2x3 design with the main factors: phenotype (Poly I:C/saline) and treatment (control/omega-3 PUFAs/NAC). On gestational day (GD) 15, the dams placed in individual cages and left to acclimatize for 2 h. Then, they were carefully anesthetized with isoflurane and injected intravenously with either the viral analogue Poly I:C (4 mg/kg; Sigma, Germany, dissolved in saline) (*n* = 24) or 0.9% saline (*n* = 24) through the tail vein (volume: 100 µL/100 g bodyweight) [25,26]. The dams were subsequently randomized into six groups: saline control, saline omega-3, saline NAC, Poly I:C control, Poly I:C omega-3, and Poly I:C NAC. No more than two rats from the same litter were used in each of the experimental groups. 

**Figure 1:**
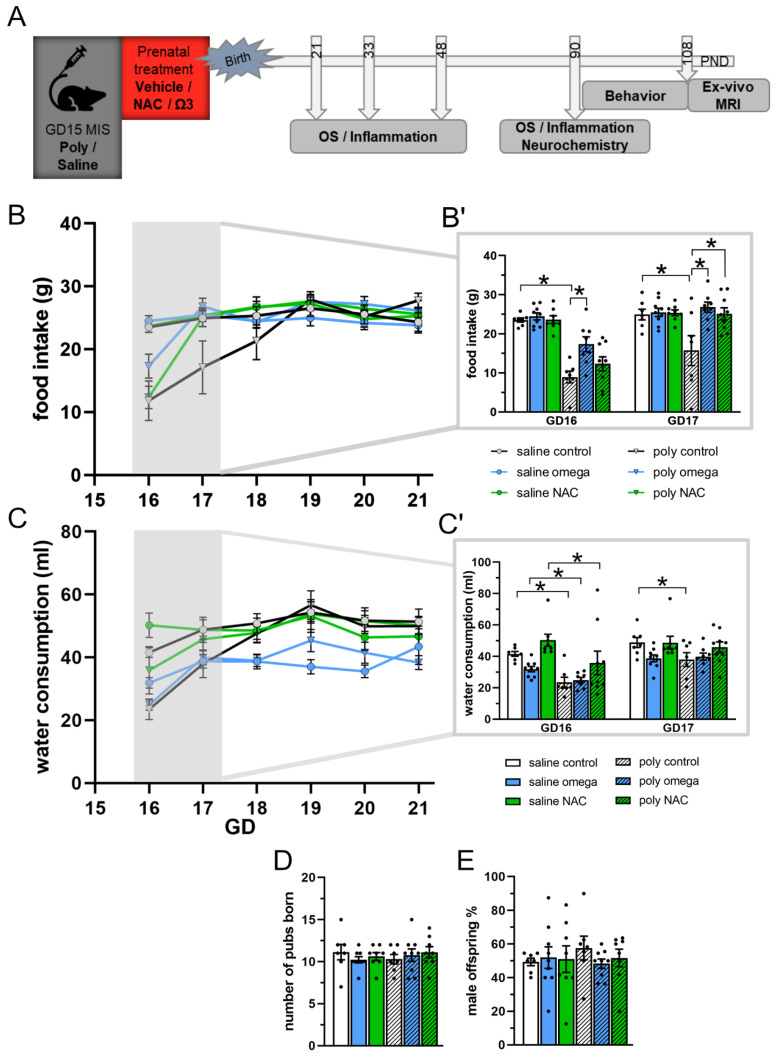
Experimental design and maternal state. (**A**) Experimental design showing the time points for phenotype induction, prenatal treatment, and subsequent testing of the offspring. (**B**,**B′**) Maternal food intake (in grams). At GD16, food consumption was significantly reduced in the dams injected with Poly I:C (post hoc test *, saline control vs. Poly I:C control: *p* < 0.001), whereas this was less pronounced in Poly I:C animals treated with omega-3 PUFAs (post hoc test *: Poly I:C control vs. Poly I:C omega-3: *p* < 0.001). At GD17, food consumption had normalized in Poly I:C animals treated with both omega-3 PUFAs and NAC, whereas the Poly: I:C controls continued to display reduced food intake (post hoc test *: saline control vs. Poly I:C control: *p* = 0.009; Poly I:C control vs. Poly I:C NAC: *p* = 0.014; Poly I:C control vs. Poly I:C omega-3: *p* = 0.003). (**C**,**C′**) Maternal water intake (in milliliters) after phenotype induction from GD16 to GD21. At GD16, water intake was significantly reduced in all Poly I:C groups following phenotype induction (post hoc test *: all Poly I:C vs. respective saline groups: *p* < 0.001). At GD17, water intake was restored in Poly I:C animals treated with omega-3 PUFAs and NAC, whereas the Poly I:C controls continued to display reduced water intake (post hoc test *: saline controls vs. Poly I:C controls: *p* = 0.009). (**D**) Number of pups born in each experimental group. (**E**) Percentage of male offspring in each experimental group. B′ shows subimage of figure B, focusing on GD16-17. C′ shows subimage of figure C, focusing on GD16-17 GD: gestational day, PND: postnatal day, OS: oxidative stress, MRI: magnetic resonance imaging, NAC: N-acetyl-cysteine (n; saline: control = 7, omega-3 = 8, NAC = 9; n Poly I:C: control = 7, omega-3 = 8, NAC = 9). Asterisks * indicate significant post hoc tests.

Male offspring from each of the six groups were assigned to different investigations, and thus randomized to either undergo behavioral experiments and MRI; post-mortem biochemical analyses at PND 21, 33, 48, or 90; or post-mortem HPLC analyses at PND 90 (Figure 1A,E and Table S1 in Supplementary Materials).

### 2.3. Supplementary Treatment during Pregnancy

The prenatal treatments were applied four hours following phenotype induction on GD15 and up until delivery, either as food supplementation (omega-3 PUFAs) or drinking water mix (NAC). This starting time for the treatment was chosen because inflammatory activity peaks between 2 and 4.5 h after the Poly I:C injection [37]. Taking into account the water quantity a pregnant dam drinks per day [38] and the target dose of 500 mg/kg body weight/day [39,40], a stock solution was prepared and mixed with tap water for a final concentration of 125 mg NAC/40 mL. Food supplemented with omega-3 PUFA fish oil was obtained from Ssniff Spezialdiäten GmbH (~18% EPA and 12% DHA). The animals received a daily supplement of 1.4 g omega-3 PUFAs/kg body weight at a concentration of 350 mg omega-3 PUFAs/20 g food [38,41]. All pregnant dams received 50 g food and 100 mL water per day.

### 2.4. Behavioral Testing

Following phenotype induction, the pregnant dam’s food and water intake was assessed daily at the same time (4 p.m.) during the first 6 days (GD16-GD21). A reduction in either food or water intake compared to the saline groups was considered a decrease in the well-being of the Poly I:C mothers. 

Behavioral testing of the offspring was related to core symptoms of schizophrenia and was carried out during the light phase, with three days between tests and in the following order: Pre-Pulse Inhibition (PPI) for sensorimotor gating, i.e., positive symptoms; Social Interaction (SI) for social behavior, i.e., negative symptoms; and Discrimination Reversal (DR) for selective attention, i.e., cognitive symptoms.

Pre-Pulse Inhibition (PPI) [26]: The PPI of the acoustic startle response (ASR) was measured in a sound-attenuating chamber (41 × 41 × 41 cm, Startle Response System, TSE, Bad Homburg, Germany) with a wire mesh cage mounted on a movement-sensitive piezoelectric measuring platform (22.5 cm × 8 cm × 8.5 cm) and two loudspeakers. The experiment consisted of a 5 min acclimatization phase and the test session. Background noise was set at 60 dB sound pressure level (SPL). During acclimatization, animals received five initial startle stimuli (100 dB SPL, white noise, 20 ms). The test session consisted of four different trial types, each delivered ten times in a pseudorandom order with an inter-trial interval of 20 to 30 s: startle pulse alone (100 dB SPL white noise, 20 ms) and three pre-pulses of 69, 73, and 81 dB (duration 30–500 ms), each followed by a startle pulse with an inter-stimulus interval of 100 ms. The ASR was calculated, and the pre-pulse trials were measured as the percentage decrease in ASR with pre-pulses. The PPI for each pre-pulse intensity was calculated according to the formula 100–100% × (mean ASR of PPI-trials/mean ASR of pulse-alone trials). 

Discrimination Reversal (DR) [42]: A T-maze was filled with water (25 °C ± 1 °C), and a hidden platform (15.5 × 15.5 cm) was placed in one arm. On day 1 (discrimination), the platform was consistently placed in one of the arms, and rats were trained to discriminate between the left/right position. If rats chose the correct arm, they could remain on the platform for 5 s. If rats chose the wrong arm, they were confined there for 5 s. Training continued until a criterion of five consecutive correct trials was reached within a maximum of 25 trials. On the next day, the rats were first retrained until the criterion on the position discrimination was reached, and then trained until reaching the criterion on the reversal of this discrimination (reversal), i.e., with the platform being located in the opposite arm. The number of trials to reach the criterion was recorded for both days. 

Social Interaction (SI) Test [43]: Prior to the experiment, rats were habituated to the testing room and arena (Plexiglas chamber, 69.5 × 68.5 × 40 cm) for 30 min. Test rats were paired with sex-, age-, and weight-matched naïve Wistar rats (actor rats). On the testing day, the rats were habituated for 30 min and marked (using finger paint) to clearly distinguish between test and actor rats. An experimental rat and its unfamiliar social partner were placed in the arena. The sessions lasted for 10 min and their behavior was recorded. The frequency of behaviors was quantified for approaching/following and the non-social control behavior of rearing. The time spent with anogenital and non-anogenital exploration was quantified. An experimenter blind to the experimental condition performed the quantification using EthoVision XT 11 software (Noldus, Wageningen, The Netherlands).

### 2.5. Post-Mortem Analysis

Animals were anesthetized with pentobarbital (i.p.,60 mg/kg), decapitated, and the brains were extracted. The prefrontal cortex (PFC), striatum (Stria), and hippocampus (Hipp) were dissected from both hemispheres [44], immediately frozen in liquid nitrogen, and stored at −80 °C. The frozen tissue samples were weighed, complemented with 1 mL ice cold Tris-HCL buffer containing 0.5% Triton X-100, and homogenized for 10–20 s with a sonicator. The homogenate was centrifuged at 10,000× *g* for 15 min and the supernatant was collected in aliquots. All sample preparation steps were carried out at 4 °C. One aliquot was used for protein quantification using the Pierce™ 660 nm Protein Assay (ThermoScientific). The remaining aliquots were stored at −80 °C until assay. 

### 2.6. Enzyme Activity

Glutathione peroxidase activity was detected using a commercial kit (GPx, abcam ab102530, colorimetric method). A volume of 25 µL of sample (containing 0.11 ± 0.4 mg protein) was diluted with 25 µL ice cold assay buffer and the GPx activity was measured in 5 min intervals at OD340 nm as the decrease in NADPH. For analysis, the data obtained at 10 min of incubation, during which enzymatic activity was evident but had not reached a plateau, was used and calculated employing a NADPH standard curve. Superoxide dismutase activity was assessed using a commercial kit (SOD; abcam ab6535,4 colorimetric method). All samples were diluted with the provided working solution for a final protein concentration of 0.5 mg/mL and the activity was measured at OD450 nm as percent inhibition. All sample measurements were taken in duplicates. The results are presented as % of controls, in which each individual value was divided by the mean of the saline control group × 100. 

### 2.7. Western Blot

Relative levels of cluster of differentiation 68 (CD68) protein in reference to the housekeeping protein beta actin were analyzed as indicators of microglia activity. The samples were loaded with Laemmli Sample Buffer (Biorad) and β-mercaptoethanol. The proteins were separated by sodium duodecyl sulfate polyacrylamide gel electrophoresis (SDS-PAGE) using Mini-Protean^®^ TGX™ Precast Gels (Biorad) and electrophoretically transferred to 0.2 µm nitrocellulose membranes. Nonspecific binding was blocked by incubation with 5% nonfat dry milk in PBS containing 0.1% Tween 20 for 1 h. The membranes were incubated with primary antibodies at 4 °C overnight. Secondary antibodies conjugated with horseradish peroxidase were applied for 1 h at room temperature. The antibodies used were mouse anti-rat beta actin (VMA00048; Biorad, 1:4000), mouse anti-rat CD68 (MCA341R; Biorad; 1:500), goat anti-mouse IgG (H + L)-HRP (STAR 117P; Biorad, 1:2000 for CD68), and donkey anti-goat IgG (H + L)-HRP (ab205723; abcam, 1:2000). The signals were detected using SuperSignal^®^ West Femto Trial Kit (Thermo scientific) on a chemiluminescence imaging system (Fusion Fx Vilber Lourmat). The generated data files were analyzed using ImageLab 6.0 software from Biorad (Copenhagen, Denmark). The results are presented as % of controls, in which each individual value was divided by the mean of the saline control group × 100.

### 2.8. HPLC

Animals were anesthetized with pentobarbital (i.p., 60 mg/kg) and decapitated; whole brains were extracted and snap-frozen for 2 min at −20 to −40 °C in methylbutane and stored at −80 °C. Unilateral samples from the medial prefrontal cortex (mPFC), hippocampus (Hipp), and striatum (Stria) were taken through 1 mm diameter micropunches and then homogenized in 250 µL 0.1 M perchloric acid by ultrasonication 3 times during 10 s while maintained on crushed ice. After protein quantification (Pierce™ 660 nm Protein Assay; ThermoScientific), dopamine (DA) and DOPAC were calculated as µg/g protein by electrochemical detection using a HPLC Agilent Platform equipped with a PRONTOSIL 120-5-C18SH analytical column. The results are presented as % of controls, in which each individual value was divided by the mean of the saline control group × 100.

### 2.9. Ex Vivo MRI 

*Image acquisition:* Following behavioral testing (~PND 114), the rats were anesthetized with pentobarbital (60 mg/kg) and transcardially perfused with PBS and cold PFA 4%. The heads were collected and refrigerated in PFA at 4 °C overnight, after which they were moved to 20% sucrose in PBS. MRI scanning was performed 3–5 days later using a 7.0 Tesla rodent scanner (Bruker BioSpin MRI GmbH, Ettlingen, Germany) at the Charité Universitätsmedizin (Berlin, Germany). For scanning, the brain was left in the skull and placed in a 50 mL Falcon tube without any solution to secure alignment and the correct orientation in anatomical space. The acquisition protocol consisted of a multi-slice localizer (field of view (FOV) 50 x50 mm) and T2-weighted contrast images with a rapid acquisition with relaxation enhancement (RARE) sequence (imaging parameters: TR/TE = 4050/30 ms, RARE factor 8, NEX 6, FOV 30 × 30 mm, MD 256 × 256) resulting in 42 slices every 0.5 mm. The total scanning time amounted to 13 min per perfused rat head. 

Lateral Ventricle volume: Extraction of lateral ventricle volumes was achieved through an in-house automated analysis platform based on the Advanced Normalization Tools and implemented on a high-performance computing setup [45]. Following DICOM to NIfTI format conversion, the images were reoriented into standard anatomical space based on the ITK-SNAP employing the orient algorithm of the Convert3D tool. All the images were reoriented into standard space using a transformation matrix defining the orientation of the voxels of the NIfTI image data sets. The initial orientation LPI (Left Posterior, Inferior) showing voxel orientation across the X, Y, Z axes, respectively, was changed to the RIP (Right, Inferior, Posterior). The N4BiasFieldCorrection algorithm was used to correct intensity nonuniformity within images. Fifty iterations over three levels with a convergence threshold of 1× 10^−6^ and full width at half-maximum deconvolution kernel of 0.15 mm were employed with default parameters. For brain extraction, the python command SkullStrip was used. As reference, a stripped image with its brain mask (manually delineated once) was used. The antsMultivariateTemplateConstruction.sh script was utilized for a within-study template from four control subjects. An iteration was employed with the greedy symmetric normalization transformation and a cross-correlation similarity metric. The anatomical image of the template was manually delineated using ITK-SNAP. The left and right hemispheres were considered as one unit. The antsRegistration command was applied. The cross-correlation similarity metric was used for diffeomorphic registration and the mutual information during linear registration between the fixed and moving images. The antsApplyTransforms command was used for label propagation of all subjects with a nearest neighbor interpolation scheme. Visual quality control was carried out to verify the quality of segmentation. The volume information was extracted using the LabelStats algorithm and MATLAB Version 9.4 (R2018a).

### 2.10. Statistical Analysis

PPI and data concerning maternal food and water intake, normalized to the dam’s weight, were analyzed using repeated measure analysis of variance (ANOVA) with phenotype, treatment, and day/pre-pulse intensity/stage as variants. The data on social interaction, brain morphology, neurochemical alterations (DA levels and DA turnover), and anti-oxidant enzymatic activity (SOD/GPx) were analyzed using 2-way ANOVA with phenotype and treatment as variants. The analyses of neurochemical alterations and enzymatic activity were split by brain region and PND, respectively. This was followed by Holm–Sidak post hoc test if applicable. DR was analyzed using non-parametric Kruskal–Wallis followed by Bonferroni corrected post hoc testing. Normality was tested using the Shapiro–Wilk Test and Q-Q Plot inspection. Equality of variances was tested using Levene’s test. For ANOVA with repeated measures, sphericity was tested using Mauchly’s Test. A *p* value < 0.05 was considered statistically significant. In the figures, significant main effects for phenotype are presented with a number sign # whereas asterisks * indicate significant post hoc tests. All statistics were calculated using SPSS 28.0.1 and figures were constructed using GraphPad Prism 8.

## 3. Results

### 3.1. Maternal State

Following phenotype induction, a decrease in food consumption was observed specifically during the first days after the dams were injected with Poly I:C (day F (5,210) = 45.13, *p* < 0.001; day × phenotype F (5,210) = 29.87, *p* < 0.001; day × treatment F (5,210) = 3.83, *p* < 0.001; day × phenotype × treatment F (5,210) = 1.99, *p* = 0.036). Post hoc testing at GD16 showed a significant omega-3 PUFA treatment effect at 24 h following phenotype induction (post hoc test, saline control vs. Poly I:C control: *p* < 0.001; Poly I:C control vs. Poly I:C omega-3: *p* < 0.001), which restored the observed reduced food consumption. At GD17, food consumption in both omega-3 PUFA and NAC treatment groups had fully normalized (post hoc test, saline control vs. Poly I:C control: *p* = 0.009; Poly I:C control vs. Poly I:C NAC: *p* = 0.014; Poly I:C control vs. Poly I:C omega-3: *p* = 0.003), while food intake was restored in untreated Poly I:C controls not before GD18- GD19 (GD18–GD21 all post hoc tests: *p* > 0.05) (Figure 1B,B′ and Table S2 in Supplementary Materials)

Similarly, water consumption was significantly decreased in all Poly I:C groups following phenotype induction and normalized more rapidly with NAC and omega-3 PUFA treatments (day F (5,210) = 38.88, *p* < 0.001; day × phenotype F (5,210) = 25.71, *p* < 0.001; day × treatment F (5,210) = 3.8006, *p*= 0.001. Post hoc test: GD16 all Poly I:C vs. respective saline groups: *p* < 0.001; GD17 saline controls vs. Poly I:C controls: *p* = 0.009) (Figure 1C and Table S2 in Supplementary Materials). The treatments did not affect the mean number of pubs being born in each group (Figure 1D,E). 

### 3.2. Oxidative and Inflammatory Parameters during Development

**Figure 2:**
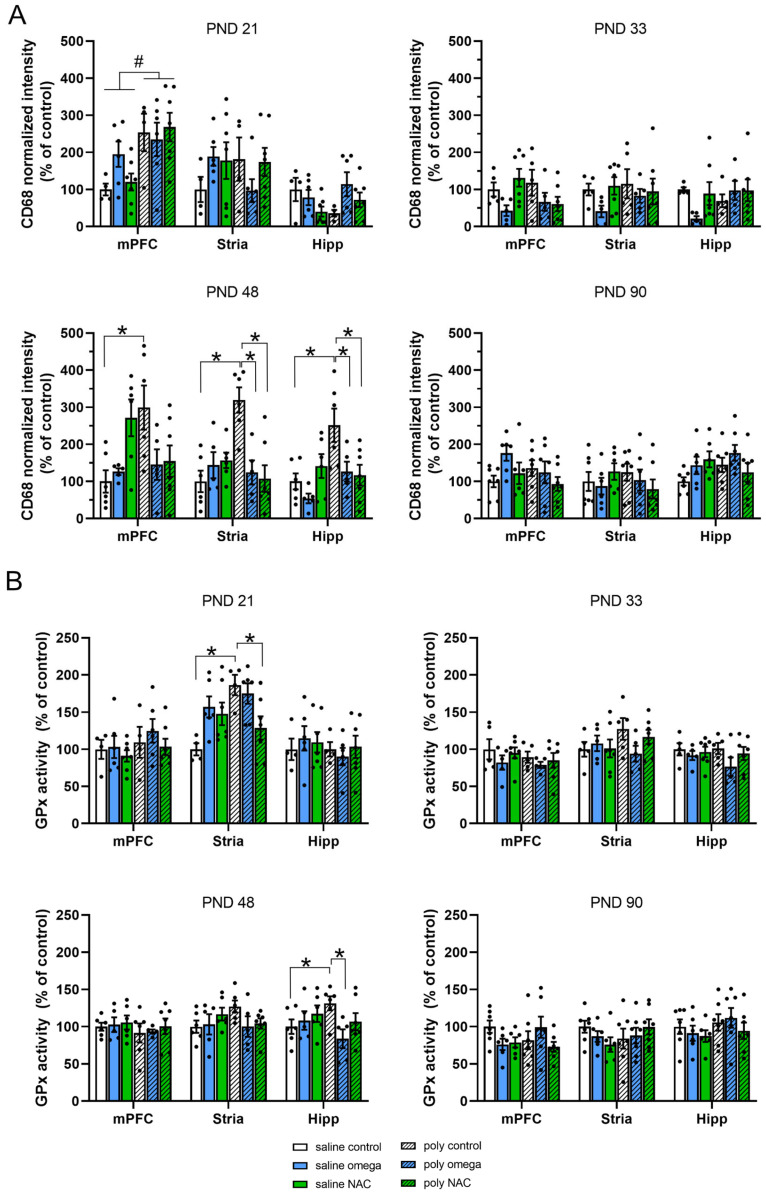
Oxidative and inflammatory parameters during development. (**A**) CD68 intensity levels normalized to controls, used as a marker of microglial activity assessed at PND 21, PND 33, PND 43, and PND 90 in the mPFC, striatum, and hippocampus. At PND 21, CD68 levels were generally increased in the mPFC of the Poly I:C animals (significant main effect # for phenotype F (1,28) = 13.667, *p* < 0.001). At PND 48, Poly I:C controls displayed a significant increase in microglial activity in the mPFC (post hoc test *: saline control vs. Poly I:C control, *p* = 0.003), the striatum (post hoc test *: saline control vs. Poly I:C control, *p* < 0.001) and the hippocampus (post hoc test *: saline control vs. Poly I:C control, *p* = 0.001). Both NAC and omega-3 PUFAs prevented the increase in microglial activity in the striatum (post hoc test *: Poly I:C control vs. Poly I:C NAC, *p* < 0.001; Poly I:C control vs. Poly I:C omega-3, *p* < 0.001) and the hippocampus (post hoc test *: Poly I:C control vs. Poly I:C NAC, *p* = 0.008; Poly I:C control vs. Poly I:C omega-3, *p* = 0.026). (**B**) GPx activity levels as % of controls used to assess activity levels of anti-oxidant enzymes at PND 21, PND 33, PND 43, and PND 90 in the mPFC, striatum, and hippocampus. At PND 21, GPx activity was significantly increased in the striatum of the Poly I:C controls (post hoc test *: saline control vs. Poly I:C control, *p* = 0.002), which was prevented by prenatal exposure to NAC (post hoc test *: Poly I:C control vs. Poly I:C NAC, *p* = 0.042). At PND 48, GPx activity was significantly increased in the hippocampus of the Poly I:C controls (post hoc test *: saline control vs. Poly I:C control, *p* = 0.05), which was prevented by omega-3 PUFA treatment (post hoc test *: Poly I:C control vs. Poly I:C omega-3, *p* = 0.020). PND: postnatal day, mPFC: medial prefrontal cortex, stria: striatum, Hipp: hippocampus, GPx: glutathione peroxidase activity, CD68: cluster of differentiation 68, NAC: N-acetyl cysteine (PND 21: n saline: control = 5, omega-3 = 6, NAC = 7; n Poly I:C: control = 5, omega-3 = 6, NAC = 7; PND 48: n saline: control = 6, omega-3 = 5, NAC = 6; n Poly I:C: control = 6, omega-3 = 5, NAC = 7; PND 33: n saline: control = 5, omega-3 = 5, NAC = 7; n Poly I:C: control = 5, omega-3 = 5, NAC = 7; PND 90: n saline: control = 7, omega-3 = 6, NAC = 6; n Poly I:C: control = 7, omega-3 = 7, NAC = 7). Number sign # indicates significant main effect for phenotype. Asterisks * indicate significant post hoc tests.

At PND 21, the CD68 levels were generally increased in the mPFC of the Poly I:C animals (phenotype F (1,28) = 13.667, *p* < 0.001). At PND 48, Poly I:C controls displayed a significant increase in microglial activity in the mPFC (phenotype × treatment F (2,29) = 7.132, *p* = 0.003; Post hoc test: saline control vs. Poly I:C control, *p* = 0.003), the striatum (treatment F (2,29) = 3.871, *p* = 0.032; phenotype × treatment F (2,29) = 10.871, *p* < 0.001; post hoc test, saline control vs. Poly I:C control, *p* < 0.001) and the hippocampus (phenotype F (1,29) = 7.102, *p* = 0.012; treatment F (2,29) = 3.754, *p* = 0.035; phenotype x treatment F (2,29) = 4.516, *p* = 0.020; post hoc test: saline control vs. Poly I:C control, *p* = 0.001). This increase in activity was prevented by prenatal exposure to both NAC and omega-3 PUFAs in the striatum (post hoc test, Poly I:C control vs. Poly I:C NAC *p* < 0.001, Poly I:C control vs. Poly I:C omega-3, *p* < 0.001) and hippocampus (post hoc test: Poly I:C control vs. Poly I:C NAC, *p* = 0.008; Poly I:C control vs. Poly I:C omega-3, *p* = 0.026) in the Poly I:C animals. No difference in microglial activity was seen across groups at PND 33 or PND 90 (Figure 2A and Table S2 in Supplementary Materials).

At PND 21, GPx activity was significantly increased in the striatum of the Poly I:C controls (phenotype F (1,28) = 5.263, *p* = 0.029; phenotype x interaction F (2,28) = 5.705, *p* = 0.008; post hoc test: saline control vs. Poly I:C control, *p* = 0.002), which was prevented by prenatal exposure to NAC (post hoc test: Poly I:C control vs. Poly I:C NAC, *p* = 0.042). At PND 48, GPx activity was significantly increased in the hippocampus of the Poly I:C controls (phenotype x treatment F (2,29) = 3.535, *p* = 0.043; post hoc test: saline control vs. Poly I:C control, *p* = 0.05), which was prevented by prenatal exposed to omega-3 PUFAs (post hoc test: Poly I:C control vs. Poly I:C omega-3, *p* = 0.020). No difference in GPx activity was seen across groups at PND 33 or PND 90 (Figure 2B and Table S2 in Supplementary Materials).

No difference in SOD activity between groups was observed at any time point during development (See Figure S1 in Supplementary Materials). 

### 3.3. Dopamine System in Adulthood

**Figure 3:**
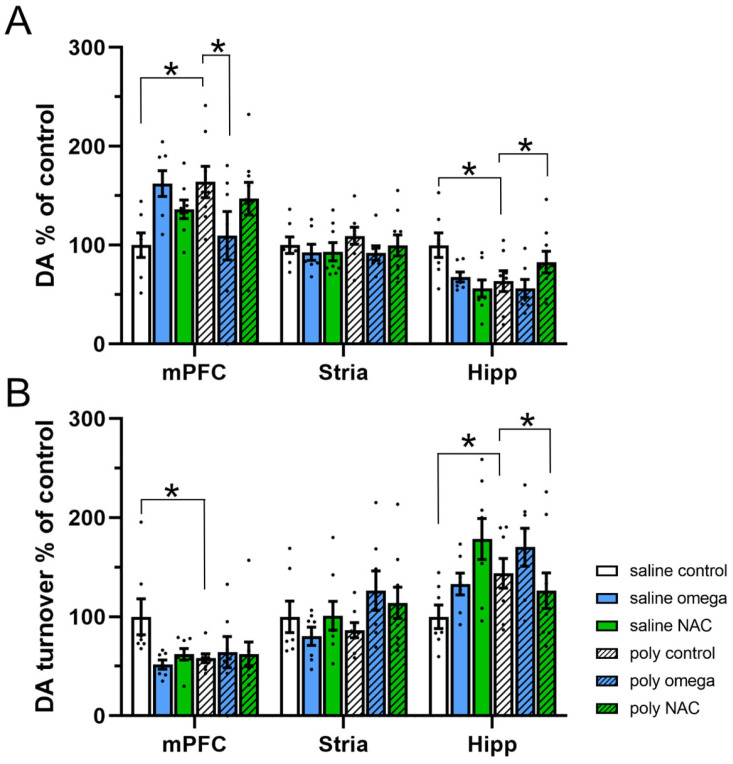
Dopamine system in adulthood (**A**) Dopamine levels expressed as % of controls across regions. Poly I:C controls displayed an increase in DA content within the mPFC (Post hoc test *: saline controls vs. Poly I:C controls: *p* = 0.008), which was significantly decreased by omega-3 (Post hoc test *: Poly I:C control vs. Poly I:C omega-3: *p* = 0.031). A reduction in DA content was also observed in the hippocampus of the Poly I:C animals (Post hoc test *: saline controls vs. Poly I:C controls: *p* = 0.014), which was increasedfollowing prenatal exposure to NAC (Post hoc test *: Poly I:C control vs. Poly I:C NAC: *p* = 0.05) (**B**) Dopamine turnover expressed as % of controls across regions. A significant decrease in DA turnover was seen in the mPFC of the Poly I:C animals (Post hoc test *: saline controls vs. Poly I:C controls: *p* = 0.013). An increase in DA turnover was seen in the hippocampus (Post hoc test *: saline controls vs. Poly I:C controls: *p* = 0.05), which was prevented following prenatal exposure to NAC (Post hoc test *: Poly I:C control vs. Poly I:C NAC: *p* = 0.026) (n saline: control = 7, omega-3 = 7, NAC = 8; n Poly I:C: control = 8, omega-3 = 7, NAC = 9). NAC: N-acetylcysteine, DA: dopamine, mPFC: medial prefrontal cortex, Stri: striatum, Hipp: hippocampus. Asterisks * indicate significant post hoc tests.

The adult offspring of Poly I:C controls displayed an increase in DA content within the mPFC (phenotype x treatment F (2,40) = 6.360, *p* = 0.004; post hoc test: saline controls vs. Poly I:C controls, *p* = 0.008), which was significantly decreased following prenatal exposure to omega-3 PUFAs (post hoc test: Poly I:C control vs. Poly I:C omega-3, *p* = 0.031). In the hippocampus, a reduction in DA content was observed (phenotype x treatment F (2,40) = 5.376, *p* = 0.009; post hoc test: saline controls vs. Poly I:C controls, *p* = 0.014), which was increased following prenatal exposure to NAC (post hoc test, Poly I:C control vs. Poly I:C NAC: *p* = 0.05). In the striatum, no differences in DA content among the groups were found (Figure 3A and Table S2 in Supplementary Materials).

In the adult offspring of Poly I:C controls, an increase in DA turnover was seen in the hippocampus (phenotype x treatment F (2,40) = 5.373, *p* = 0.009; post hoc test: saline controls vs. Poly I:C controls, *p* = 0.05), which was prevented following prenatal exposure to NAC (post hoc test: Poly I:C control vs. Poly I:C NAC, *p* = 0.026). The decrease in DA turnover was not affected by either treatment within the mPFC (phenotype x treatment F (2,40) = 3.103, *p* = 0.056; post hoc test: saline controls vs. Poly I:C controls, *p* = 0.013) (Figure 3B and Table S2 in Supplementary Materials).

### 3.4. Behaviors in Adulthood

**Figure 4:**
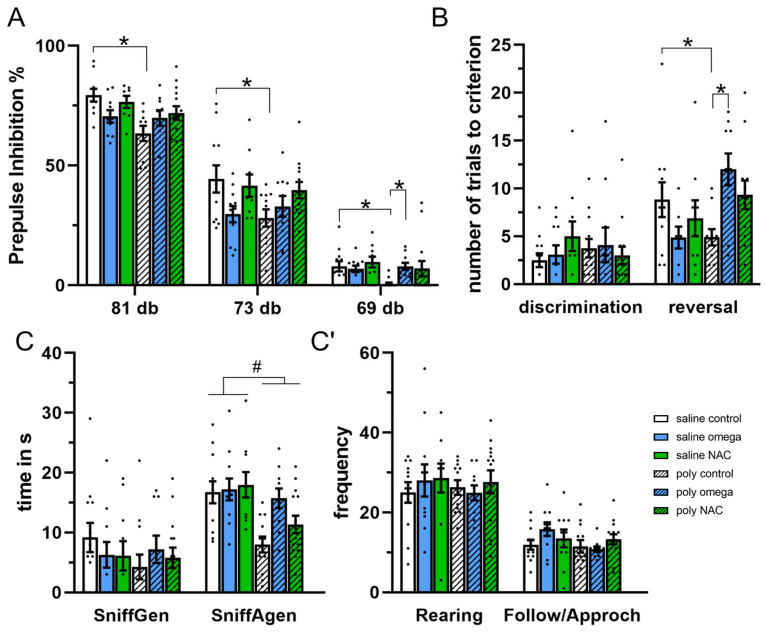
Behaviors in adulthood (**A**) Pre-pulse inhibition paradigm, displaying percentage pre-pulse inhibition at 81, 73 and 69dB. Poly I:C controls displayed deficits in all pre-pulses tested (Post hoc test *: saline control vs. Poly I:C control: 81dB *p* < 0.001, 73 dB *p* = 0.021, 69 dB *p* = 0.012). Exposure to omega-3 improved the pre-pulse deficits seen for 69dB in the Poly I:C animals (Post hoc test *: Poly I:C control vs. Poly I:C omega-3: 69dB *p* < 0.05).(n saline: control = 12, omega-3 = 11, NAC = 10; n Poly I:C: control = 11, omega-3 = 10, NAC = 14). (**B**) Discrimination reversal paradigm, displaying number of trials needed to reach the criterion for discrimination and reversal testing. Poly I:C control rats showed rapid reversal (Post hoc test *: saline control vs. Poly I:C controls *p* = 0.05), which was significantly improved following exposure to omega-3 (Post hoc test *: Poly I:C controls vs Poly I:C omega-3, *p* = 0.045) (n saline: control = 11, omega-3 = 8, NAC = 9; n Poly I:C: control = 11, omega-3 = 10, NAC =12). (**C**,**C′**) Social interaction paradigm displaying the time spent in seconds sniffing genitals (Sniffgen) and anogenital (SniffAgen), as well as frequency of rearing and follow/approach behavior Poly I:C rats displayed a general decrease in social interactions, by means of reduced anogenital sniffing (significant main effect # for phenotype F (1,63) = 16.18, *p* < 0.001) (n saline: control = 12, omega-3 = 11, NAC = 10; n Poly I:C: control = 12, omega-3 = 10, NAC = 14). dB: decibel, s: seconds. Number signs # indicates significant main effect for phenotype. Asterisks * indicate significant post hoc tests.

The adult offspring of Poly I:C rats displayed deficits in pre-pulse inhibition (pre-pulse F (2,106) = 1093, *p* < 0.001; phenotype F (1,53) = 4.916, *p* = 0.031; phenotype x treatment, F (2,53) = 3.707, *p* = 0.030. Post hoc test: saline control vs. Poly I:C control: 81 dB *p* < 0.001, 73 dB *p* = 0.021, 69 dB *p* = 0.012), which was improved following prenatal exposure to omega-3 PUFAs (post hoc test: Poly I:C control vs. Poly I:C omega-3 PUFAs: 69 dB *p* < 0.05) (Figure 4A and Table S2 in Supplementary Materials).

In the DR paradigm, no difference in discrimination (day 1) across groups was found. A significant difference in reversal learning was found across groups (day 2, H (5) = 13.506, *p* = 0.019), as the adult offspring of Poly I:C rats showed a trend towards rapid reversal (post hoc *p* = 0.051), indicative of excessive switching behavior, which was significantly improved following prenatal exposure to omega-3 PUFAs (post hoc *p* = 0.045) (Figure 4B and Table S2 in Supplementary Materials). 

The adult offspring of Poly I:C rats displayed a general decrease in social interactions, measured as reduced anogenital sniffing (phenotype F (1,63) = 16.18, *p* < 0.001), which was not affected by supplement treatment (Figure 4C, and Table S2 in Supplementary Materials).

### 3.5. Brain Structure in Adulthood

**Figure 5:**
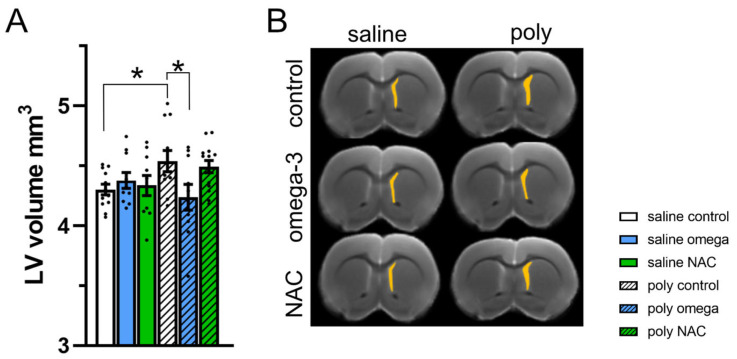
Size of lateral ventricles in adulthood. (**A**) The size of the lateral ventricles in mm^3^ across groups. Poly I:C controls displayed enlarged lateral ventricles (post hoc test *: saline controls vs. Poly I:C controls, *p* = 0.025), which was prevented following exposure to omega-3 PUFAs (post hoc test *: Poly I:C control vs. Poly I:C omega-3, *p* = 0.020) (**B**) Ex vivo MRI visualizing the volume of the lateral ventricles (mm^3^) (n saline: control = 12, omega-3 = 11, NAC = 10; n Poly I:C: control = 12, omega-3 = 10, NAC = 14), LV: lateral ventricle, NAC: N-acetyl-cysteine. Asterisks * indicate significant post hoc tests.

The adult offspring of Poly I:C controls displayed enlarged lateral ventricles (phenotype x treatment F (2,61) = 3.43, *p* = 0.039; post hoc test: saline controls vs. Poly I:C controls, *p* = 0.025), which was prevented by prenatal exposure to omega-3 PUFAs (post hoc test: Poly I:C control vs. Poly I:C omega-3, *p* = 0.020) (Figure 5A,B and Table S2 in Supplementary Materials)

## 4. Discussion

### 4.1. Supplementary Treatment Affected the Inflammatory Response in Both the Dams and Their Offspring

We found that exposure to NAC and omega-3 PUFAs benefitted the mothers as well as the offspring, who, once reaching adulthood, displayed partial relief in schizophrenia-related outcomes. Several studies have shown that microglial alterations in the Poly I:C model are correlated with schizophrenia-like symptoms [46,47,48,49,50]. This aligns with clinical findings indicating an important role of microglial alterations in the immune dysregulation observed in patients with schizophrenia [51]. As indicated by the CD68 upregulation, we found a pronounced increase in microglial activity starting in the mPFC at PND 21, which was absent at PND 33, and then detected again in the mPFC, striatum, and hippocampus once the Poly I:C control rats reached adolescence (PND 48). It remains to be investigated what triggers this re-activation in microglia activity, now affecting all brain regions, in the adolescent animals. It might indicate an alteration in the readiness of the microglia, which are kept latent in the earlier stages of life, only to later become active. Indeed, it is suggested that in schizophrenia, a subset of microglia may be maintained in a permanent primed state as a consequence of prenatal infection, which later serves as a vulnerability factor for an increase in cytokine release and subsequent changes in cognition and affective behavior [52,53,54]. At PND 21, microglial activation in the mPFC was increased across all Poly I:C groups. The evolution of schizophrenia is found to rely on time-dependent alterations within interconnected brain structures, with alterations in the mPFC suggested as an early sign [55,56,57,58]. It remains to be investigated whether the early increase in microglial activation, specifically within the mPFC, also reflects an early signal of an emerging disorder. Adolescence and early adulthood are recognized as vulnerable time periods in schizophrenia, and may, in some individuals, constitute a prodromal phase before the first psychotic symptoms [59]. Similar to our findings, others have shown that microglial alterations in Poly I:C rats tend to arise between periadolescence (PND 35) and early adulthood (PND 74), which occurs in the hippocampus, striatum, and prefrontal cortex [46,60]. In schizophrenia, a further recruitment of subcortical brain areas in particular as the disease progresses is thought to eventually set the ground for the full manifestation of symptoms [57,58]. As such, we suggest that the re-activation of microglia across interconnected pathology-relevant brain areas in the adolescent Poly I:C rats may mark a transition towards disease manifestation. The prevention of later symptoms through early intervention has shown to rely on a modulation of the preexisting abnormal microglial properties in the Poly I:C model [47,50]. Prenatal exposure to both NAC and omega-3 PUFAs prevented the surge in microglia activation in the adolescent offspring, especially within the striatum and hippocampus, which may be linked to both supplements also partially relieving some of the symptoms later found in the adult Poly I:C animals. Both NAC and omega-3 PUFAs were also demonstrated to benefit the dams. Exposure to Poly I:C immediately decreased the intake of food and water, reflecting a reduction in the well-being of the dams. Both NAC and omega-3 PUFAs led to a faster restoration of normal consumption levels compared to the Poly I:C control rats who eventually got better over time without any intervention. This accelerated improvement may be attributed to the anti-inflammatory properties of both supplements, counteracting the heightened inflammatory response in the mothers following exposure to the virus-mimicking compound [37]. However, the potential direct effects on the fetus need further assessment. 

### 4.2. Prenatal Supplementary Treatment Partially Prevented Deregulation in the Collective Enzyme Anti-Oxidant Defense System in the Developing Offspring

The activity of the anti-oxidant enzymes SOD and GPx throughout development was used as an indirect measure of the oxidative status in the Poly I:C model. The major anti-oxidant enzymes are critical at different stages for removing free radicals, as SOD catalyzes the dismutation of ROS to hydrogen peroxide (H_2_O_2_), which is then removed by GPx [61]. Alteration in one of these enzymes without compensation in the other increases the risk of redox deregulation and oxidative damage [62]. Within the post-mortem brains of patients with schizophrenia, low levels of GPx have been found in the caudate putamen, whereas high levels of SOD have been observed in the frontal cortex alongside normal levels of this enzyme in the putamen, thalamus, and caudate nucleus [62,63]. This is inconsistent with our results, as we did not, at any time point, observe changes in SOD activity in any of the brain areas investigated, and rather an increase in GPx activity was seen in the striatum at PND 21 and again in the hippocampus at PND 48 in the offspring of the Poly I:C group. It is worth mentioning that the proliferation of microglial is especially mediated by H_2_O_2_, which is the substrate for GPx [64]. Thus, further studies are needed to evaluate whether the increase in GPx activity found in the Poly I:C controls is related to the increase in microglial activity found during the same time periods. Nevertheless, the mismatch between normal SOD activity, alongside changes in GPx in the Poly I:C controls indicates a potential deregulation in the collective enzymatic anti-oxidant defense system, which theoretically may affect the oxidative status in these animals. Indeed, it has been shown that neonatal Poly I:C exposure results in oxidative damage at PND 74 by means of reduced glutathione and increased lipid peroxidation [60]. Prenatal exposure to NAC reduced GPx activity in the striatum at PND 21, whereas GPx activity in the hippocampus at PND 48 was reduced following omega-3 PUFA exposure. Further studies investigating the level of oxidative stress in these animals and the subsequent impact of prenatal exposure to NAC and omega-3 PUFAs are needed. 

### 4.3. Prenatal Supplementary Treatment Partially Prevented Schizophrenia-Related Outcomes in The Adult Offspring

Alterations in the dopamine system, including an imbalance between cortical and subcortical levels, have long been considered to be involved in the pathophysiology and symptoms of schizophrenia [65,66]. In the adult offspring of the Poly I:C controls, we found an increase in DA levels alongside a decrease in DA turnover of the mPFC, and a decrease in DA levels of the hippocampus [26]. Prenatal exposure to omega-3 PUFAs prevented the increase in DA levels within the mPFC, whereas the decrease in DA levels in the hippocampus was prevented by NAC. We further found that prenatal exposure to omega-3 led to partial relief of later symptoms, mainly affecting some aspects related to positive symptoms as assessed in the PPI paradigm and cognition as reflected by rapid reversal in the Poly I:C controls. Strikingly, prenatal exposure to omega-3 was able to prevent the enlargement of lateral ventricles that was otherwise observed in the Poly I:C model. The ability to prevent the otherwise enlarged lateral ventricles in the Poly I:C model has been previously reported following preventive interventions applied during adolescence, prior to symptom manifestation [28,29,32,33,34,35]. 

### 4.4. Limitations

Our results are limited to a distinct animal model, in which the phenotype is fully present in all Poly I:C offspring. Future clinical research will be challenged by the ability to identify the group of high-risk individuals who may benefit from supplement treatment at the fetal stage. The clinical symptoms of schizophrenia are complex and, despite the Poly I:C model being well-validated, there are currently no animal models which fully mimic the human disorder. As our results only showed partial relief in distinct behaviors and pathophysiological processes, additional investigations incorporating other animal models and further behavioral paradigms are needed before any translational conclusions can be made. Further studies on the mechanism of action related to the prevention of schizophrenia-like outcomes in the offspring are also warranted.

## 5. Conclusions

Taken together, we showed that prenatal exposure to supplements known to have anti-inflammatory properties improved the well-being of the dams exposed to immune activation, and benefitted the offspring by partially improving some of the neurobiological and behavioral abnormalities otherwise found in the Poly I:C model. Thus, our results show that intake of over-the-counter supplements may assist in specifically targeting the inflammatory response related to schizophrenia pathophysiology, aiding in diminishing later disease severity in the offspring.

## Data Availability

The data sets generated and/or analyzed in the current study are available from the corresponding author upon reasonable request.

## Supplementary Materials

The following supporting information can be downloaded at: https://www.mdpi.com/article/10.3390/antiox12051068/s1, Figure S1: Oxidative and inflammatory parameters during development. Table S1: The number of male offsprings assigned to the different investigations. Table S2: Overview of the ANOVA effects.
