# Supplementary material for: Supplement Treatment with NAC and Omega-3 Polyunsaturated Fatty Acids during Pregnancy Partially Prevents Schizophrenia-Related Outcomes in the Poly I:C Rat Model
Source: Antioxidants (Basel). 2023 May 9;12(5):1068. doi: 10.3390/antiox12051068 (PMC10215079; doi:10.3390/antiox12051068)
Supplement: Supplementary file 1 [file antioxidants-12-01068-s001.zip › antioxidants-2316179-supplementary.pdf]

Supplementary Treatment with NAC and Omega-3 during Pregnancy Partially Prevents Schizophrenia-Related Outcomes in the Offspring of the Poly I:C Rat Model

Supplementary information

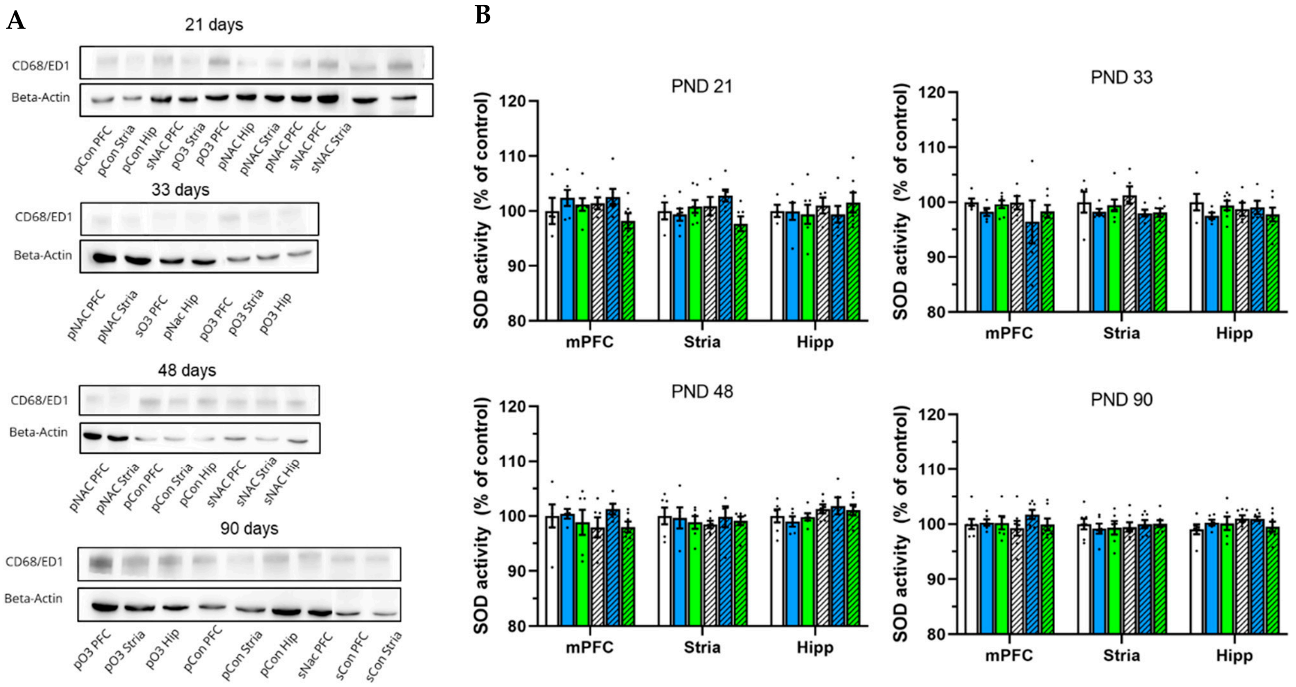

Figure S1. Oxidative and inflammatory parameters during development A) Western blot of CD68 intensity levels in reference to huosekeeping protein beta actin, used as a marker of microglial activity assessed at PND 21, PND 33, PND 43 and PND 90 in the mPFC, striatum and hippocampus B) SOD activity levels as % of controls used to assess activity levels of anti-oxidant enzymes at PND 21, PND 33, PND 43 and PND 90 in the mPFC, striatum and hippocampus. PND: postnatal day, mPFC: medial prefrontal cortex, stria: striatum, Hipp: hippocampus, SOD: superoxide dismutase activity, CD68: cluster of differentiation 68, NAC: N-acetyl cysteine, PFC: prefrontal cortex (PND 21: n saline: control = 5, omega-3 = 6, NAC = 7; n Poly I:C: control = 5, omega-3 = 6, NAC =7; PND 48: n saline: control = 6, omega-3 = 5, NAC = 6; n Poly I:C: control = 6, omega-3 = 5, NAC =7; PND 33: n saline: control = 5, omega-3 = 5, NAC = 7; n Poly I:C: control = 5, omega-3 = 5, NAC =7; PND 90: n saline: control = 7, omega-3 = 6, NAC = 6; n Poly I:C: control = 7, omega-3 = 7, NAC =7).

Table S1. The number of male offsprings assigned to the different investigations, PND: post natal day, HPLC; high performance liquid chromatography, NAC: N-acetyl cysteine, MRI: Magnetic resonance imaging.

	**Saline**			**Poly I:C**		
	Control	Omega-3	NAC	Control	Omega-3	NAC
Behavior and MRI	12	11	10	12	10	14
Biochemical evaluation						
PND 21	5	6	7	5	6	7
PND 33	5	5	7	5	5	7
PND 48	6	5	6	6	5	7
PND 90	7	6	6	7	7	7
HPLC						
PND 90	7	7	8	8	7	9

Table S2. Overview of the ANOVA effects, including degree of freedom (DF), F-values and statistical p-values. Results presented for A) maternal state, B) CD68 activity, C) GPx activity, D) dopamine content and turnover and E) behavior and lateral ventricles. RM: repeated measures, DF: degree of freedom, PND: post natal day, mPFC: medial prefrontal cortex, stria: striatum, Hipp: hippocampus, DA: dopamine, CD68: cluster of differentiation 68, GPx: Glutathione peroxidase activity.

A

**Maternal state**		**RM ANOVA effects**	**DF**	**F-value**	**p-value**
	Food intake	Phenotype	1,42	3.40	0.072
		treatment	2,42	1.13	> 0.05
		day	5, 210	45.13	< 0.001
		phenotype x treatment	2,42	1.57	> 0.05
		day x phenotype	5, 210	29.87	< 0.001
		day x treatment	5, 210	3.83	< 0.001
		day x phenotype x treatment	5, 210	1.99	0.036
	Water intake	Phenotype	1,42	2.34	>0.05
		treatment	2,42	0.64	>0.05
		day	5, 210	38.88	< 0.001
		phenotype x treatment	2,42	0.67	>0.05
		day x phenotype	5, 210	25.71	< 0.001
		day x treatment	5, 210	38.006	0.001
		day x phenotype x treatment	5, 210	1.65	0.093

B

**CD68 activity**	**PND**	**Area**	**2-way ANOVA effect**	**DF**	**F-value**	**p-value**
	21	mPFC	phenotype	1,28	13.667	< 0.001
			treatment	2,18	0.474	0.628
			phenotype x treatment	2,18	1.551	0.230
		Stria	phenotype	1,28	0.019	0.891
			treatment	2,18	0.500	0.612
			phenotype x treatment	2,18	1.950	0.161
		Hipp	phenotype	1, 28	0.004	0.948
			treatment	2,18	1.850	0.176
			phenotype x treatment	2,18	2.523	0.098
	33	mPFC	phenotype	1,28	0.248	0.623
			treatment	2,18	2.622	0.90
			phenotype x treatment	2,18	2.775	0.08
		Stria	phenotype	1,28	0.383	0.541
			treatment	2,18	1.489	0.243
			phenotype x treatment	2,18	0.556	0.580
		Hipp	phenotype	1, 28	0.800	0.379
			treatment	2,18	1.004	0.379
			phenotype x treatment	2,18	2.122	0.139
	48	mPFC	phenotype	1,29	0.904	0.349
			treatment	2,29	1.681	0.204
			phenotype x treatment	2,29	7.132	0.003
		Stria	phenotype	1,29	3.638	0.066

			treatment	2,29	3.871	0.032
			Phenotype x treatment	2,29	10.871	<0.001
		Hipp	phenotype	1,29	7.102	0.012
			treatment	2,29	3.754	0.035
			Phenotype x treatment	2,29	4.516	0.020
	90	mPFC	phenotype	1,34	0.637	0.430
			treatment	2,34	1.850	0.173
			phenotype x treatment	2,34	2.019	0.148
		Stria	phenotype	1,34	0.009	0.924
			treatment	2,34	0.230	0.796
			Phenotype x treatment	2,34	1.230	0.305
		Hipp	phenotype	1,34	0.722	0.401
			treatment	2,34	1.685	0.201
			Phenotype x treatment	2,34	2.141	0.133

C

GPx activity	PND	Area	2-way ANOVA effect	DF	F-value	p - value
	21	mPFC	phenotype	1,28	1.627	0.213
			treatment	2,28	0.883	0.425
			phenotype x treatment	2,28	0.110	0.896
		Stria	phenotype	1,28	5.263	0.029
			treatment	2,28	2.183	0.132
			phenotype x treatment	2,28	5.705	0.008
		Hipp	phenotype	1,28	0.713	0.406
			treatment	2,28	0.095	0.909
			phenotype x treatment	2,28	0.374	0.691
	33	mPFC	phenotype	1,28	1.067	0.310
			treatment	2,28	1.078	0.354
			phenotype x treatment	2,28	0.095	0.910
		Stria	phenotype	1,28	1.057	0.313
			treatment	2,28	0.557	0.579
			phenotype x treatment	2,28	1.568	0.226
		Hipp	phenotype	1,28	0.025	0.876
			treatment	2,28	1.287	0.292
			phenotype x treatment	2,28	0.844	0.441
	48	mPFC	phenotype	1,29	0.957	0.336
			treatment	2,29	0.335	0.718
			phenotype x treatment	2,29	0.026	0.974
		Stria	phenotype	1,29	0.240	0.628
			treatment	2,29	0.759	0.477
			Phenotype x treatment	2,29	2.434	0.105
		Hipp	phenotype	1,29	0.020	0.889
			treatment	2,29	1,62	0.215

			Phenotype x treatment	2,29	3.535	0.043
	90	mPFC	phenotype	1,34	0.002	0.966
			treatment	2,34	1.268	0.294
			phenotype x treatment	2,34	2.260	0.120
		Stria	phenotype	1,34	0.132	0.719
			treatment	2,34	0.123	0.885
			Phenotype x treatment	2,34	1.910	0.164
		Hipp	phenotype	1,34	1.597	0.215
			treatment	2,34	0.720	0.494
			Phenotype x treatment	2,34	0.285	0.754

D

DA Content	Area	2-Way ANOVA	DF	F-value	p-Value
	mPFC	phenotype	1,40	0.318	0.576
		treatment	2,40	0.200	0.820
		phenotype x treatment	2,40	6.360	0.004
	Stria	phenotype	1,40	0.455	0.504
		treatment	2,40	0.909	0.411
		phenotype x treatment	2,40	0.157	0.855
	Hipp	phenotype	1,40	0.777	0.383
		treatment	2,40	1.939	0.175
		phenotype x treatment	2,40	5.376	0.009
**DA turnover**	mPFC	phenotype	1,40	1.081	0.305
		treatment	2,40	1.889	0.164
		phenotype x treatment	2,40	3.103	0.056
	Stria	phenotype	1,40	1.646	0.207
		treatment	2,40	0.530	0.593
		phenotype x treatment	2,40	2.007	0.148
	Hipp	phenotype	1,40	0.495	0.486
		treatment	2,40	2.121	0.133
		phenotype x treatment	2,40	5.373	0.009

E

Behavior		RM ANOVA	DF	F-value	p-value
PPI		Prepulse	2, 106	1093	<0.001
		Phenotype	1, 53	4.916	0.031
		Treatment	2, 53	1.645	>0.05
		phenotype x treatment	2, 53	3.707	0.030
		prepulse x treatemnt	4, 106	1.73	>0.05
DR paradigm		**Kruskal-Wallis**			
	Discrimination	group	5	2,969	0,705
	Reversal	group	5	13,506	0,19
Social interaction		**2- way ANOVA**			
	SniffAgen	phenotype	1, 63	16.18	<0.001
		treatment	2, 63	2.88	0.063
		phenotype x treatment	2, 63	2.311	0.108

	SniffGen	phenotype	1, 63	0.647	0.424
		treatment	2, 63	0.086	0.918
		phenotype x treatment	2, 63	1.008	0.371
	Follow/approach	phenotype	1, 63	2.342	0.131
		treatment	2, 63	0.819	0.445
		phenotype x treatment	2, 63	1.560	0.218
	Rearing	phenotype	1, 63	0.154	0.696
		treatment	2, 63	0.394	0.676
		phenotype x treatment	2, 63	0.275	0.761
**Lateral ventricles**		**2-way ANOVA**			
		phenotype	1, 61	1.95	0.168
		treatment	2, 61	1.38	0.258
		phenotype x treatment	2, 61	3.43	0.039
